# End-Stage Achalasia With Megaesophagus Refractory to Two Heller Myotomies

**DOI:** 10.7759/cureus.55721

**Published:** 2024-03-07

**Authors:** Lorraine I Chong Tai, Omar Akil, Kimberly Q Nguyen, Aryama Sharma

**Affiliations:** 1 Internal Medicine, Broward Health Medical Center, Fort Lauderdale, USA; 2 Gastroenterology, Broward Health North, Deerfield Beach, USA

**Keywords:** esophageal achalasia, heller myotomy, mega-esophagus, severe achalasia, achalasia cardia

## Abstract

Achalasia is a motility disorder of the esophagus in which the lower esophageal sphincter fails to relax. Megaesophagus is a rare complication of achalasia characterized by severe dilatation of the esophagus, often indicative of end-stage achalasia. Typical presenting symptoms include dysphagia, nausea, vomiting, weight loss, and chest pain. The majority of patients with achalasia typically have excellent outcomes after surgical intervention with Heller myotomy. We discuss an interesting case of unsuccessful surgical intervention and hypothesize the reason for its failure in our patient.

## Introduction

Achalasia or achalasia cardia is an uncommon esophagus disease characterized by the inability of the lower esophageal sphincter (LES) to relax and the absence of peristalsis [1]. This failure of relaxation is thought to be caused by the degeneration of inhibitory neurons of the myenteric plexus [2]. Achalasia is most commonly diagnosed in those between 25 and 60 years of age with no predilection for either sex [3]. The exact etiology of this condition is unknown, although some speculate it may be related to an autoimmune, inflammatory, or infectious process, such as in Chagas disease after infection with the parasite Trypanosoma cruzi [4]. We present a case of a 64-year-old male found to have a megaesophagus secondary to severe achalasia despite undergoing surgical intervention on two separate occasions for his condition.

## Case presentation

A 64-year-old male presented to the emergency department with a one-month history of persistent nausea and vomiting with the inability to tolerate solids and liquids. Due to these symptoms, he endorsed associated fatigue and an unintentional 10-pound weight loss in the last month. He denied any fever, chills, chest pain, abdominal pain, diarrhea, or constipation. His past medical history was remarkable for polysubstance abuse with cocaine and cannabis, achalasia diagnosed in 2008, and a history of multiple endoscopies with Botox. He also underwent an unsuccessful Heller myotomy and Nissen fundoplication in 2008 and again in 2020. He previously had a percutaneous endoscopic gastrostomy (PEG) tube placed due to his achalasia, but it was removed before his repeat Heller myotomy and Nissen fundoplication in 2020. 

**Figure 1:**
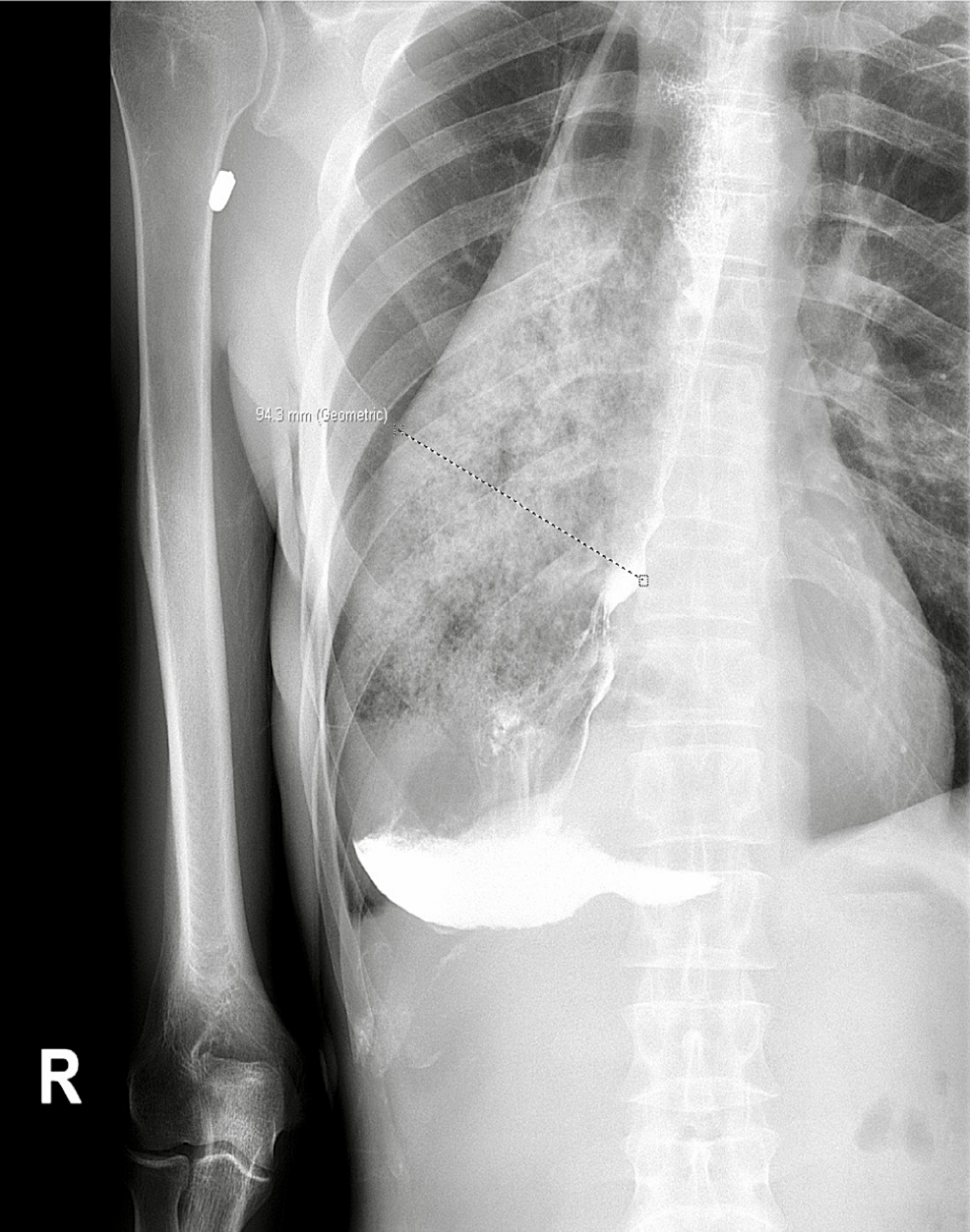
Esophagram showing a 94.3 mm in diameter severely dilated esophagus with distal esophageal narrowing compatible achalasia

An esophagram revealed a severely dyskinetic and severely distended esophagus, 94.3 mm in diameter, with distal esophageal narrowing compatible with the patient’s history of achalasia. The esophagus was filled with fluid and debris, with no evidence of emptying into the stomach throughout the study, suggesting distal esophageal obstruction (Figure 1).

Laboratory results were only remarkable for mild hypokalemia of 3.4 (normal serum potassium levels for adults: 3.5-5.2 mEq/L). He was started on a clear liquid diet and given maintenance intravenous fluids with normal saline. He was evaluated by the gastroenterologist, after which PEG tube placement was recommended, given the severity of his symptoms. He ultimately refused PEG placement and was discharged the next day with recommendations to follow up at a specialty center for surgical evaluation for either a peroral endoscopic myotomy (POEM), Ivor Lewis esophagectomy, or a redo laparoscopic Heller myotomy. He was discharged on pantoprazole 40 mg BID (twice daily) and Zofran 4 mg q8h (every 8h). Unfortunately, the patient did not follow up for surgical evaluation and refused any repeat surgical or endoscopic intervention.

## Discussion

Megaesophagus results from chronic dilatation of the esophagus [2]. Megaesophagus has been defined as a maximum esophageal diameter of more than or equal to 8 cm seen on barium esophagram [5]. An estimated 5% of patients with achalasia will develop megaesophagus and need surgical intervention [6].

Initial imaging for suspected achalasia may include a barium esophagram, which typically shows esophageal dilation and narrowing at the gastroesophageal junction (GEJ), creating a ‘bird’s beak’ sign [7]. The diagnosis of achalasia is ultimately confirmed by high-resolution esophageal manometry (HREM), demonstrating an absence of peristaltic contractions and incomplete relaxation of the LES after swallowing [7].

Traditionally, treatment options have included endoscopic intervention via endoscopic botulinum toxin injection to LES or pneumatic dilation [8]. If unsuccessful, medical management with calcium channel blockers or long-acting nitrates is considered [8]. Finally, surgical intervention via surgical myotomy or esophagectomy may be required, especially in end-stage disease (those who have developed a megaesophagus) [8]. 

Most patients with achalasia were found to have good to excellent long-term outcomes following Heller myotomy with semifundoplication [9,10]. Most symptomatic failures typically occur within the first year of surgery [10]. It is estimated that 5% of patients will need another surgical intervention for achalasia [11]. Early recurrences are hypothesized to result from incomplete myotomy, and late recurrences result from fibrosis after the myotomy or megaesophagus, as in our case [12]. Patients who had severe preoperative dysphagia, lower LES pressures, severe dilation of the esophagus, and prior endoscopic dilation or botulin toxin treatment were found to have worse surgical outcomes [13].

Esophagectomy with a hybrid Ivor-Lewis approach is only considered for those with end-stage achalasia and/or those who have undergone multiple prior surgeries in whom a redo Heller myotomy was considered futile [14]. Another option may be peroral endoscopic myotomy (POEM), which is considered to be the endoscopic equivalent of the Heller myotomy [15]. POEM is becoming the treatment of choice for achalasia and is utilized for prior failed achalasia treatment, including laparoscopic surgical myotomy, as well as for those who are considered poor surgical candidates [16]. The Yokohama group reported that 10 patients with persistent dysphagia after Heller myotomy or pneumatic dilation who later underwent POEM had significant improvement in symptoms and lower LES resting pressures [17].

Recent literature proposes that endoscopic botulinum toxin therapy may lead to obliteration of the sub-mucosal plane secondary to inflammation from repeated injections, making surgical myotomy or POEM more challenging [18-20]. This may explain why surgery was unsuccessful in our patient, given he had a history of multiple endoscopic botulinum toxin treatments.

## Conclusions

Megaesophagus is a rare complication of achalasia. Achalasia refractory to initial surgical intervention is very uncommon, much less after two surgeries, as in our case. This case highlights that these patients still require monitoring for the recurrence of symptoms. This case report also proposes that patients who continue to fail endoscopic therapy with botox injections should undergo surgical intervention sooner rather than later, given the potential complications posed to surgical outcomes with repeat injections. After an unsuccessful Heller myotomy, patients should be evaluated by a surgical specialist to undergo peroral endoscopic myotomy (POEM), Ivor Lewis esophagectomy, or a redo laparoscopic Heller myotomy.
